# An Overview on the Effect of Severe Plastic Deformation on the Performance of Magnesium for Biomedical Applications

**DOI:** 10.3390/ma16062401

**Published:** 2023-03-17

**Authors:** Mariana P. Medeiros, Debora R. Lopes, Megumi Kawasaki, Terence G. Langdon, Roberto B. Figueiredo

**Affiliations:** 1 Department of Metallurgical and Materials Engineering, Universidade Federal de Minas Gerais, Belo Horizonte 31270-901, MG, Brazil; 2 School of Mechanical, Industrial and Manufacturing Engineering, Oregon State University, Corvallis, OR 97331, USA; 3 Materials Research Group, Department of Mechanical Engineering, University of Southampton, Southampton SO17 1BJ, UK

**Keywords:** biomaterials, corrosion, magnesium, mechanical properties, severe plastic deformation

## Abstract

There has been a great interest in evaluating the potential of severe plastic deformation (SPD) to improve the performance of magnesium for biological applications. However, different properties and trends, including some contradictions, have been reported. The present study critically reviews the structural features, mechanical properties, corrosion behavior and biological response of magnesium and its alloys processed by SPD, with an emphasis on equal-channel angular pressing (ECAP) and high-pressure torsion (HPT). The unique mechanism of grain refinement in magnesium processed via ECAP causes a large scatter in the final structure, and these microstructural differences can affect the properties and produce difficulties in establishing trends. However, the recent advances in ECAP processing and the increased availability of data from samples produced via HPT clarify that grain refinement can indeed improve the mechanical properties and corrosion resistance without compromising the biological response. It is shown that processing via SPD has great potential for improving the performance of magnesium for biological applications.

## 1. Introduction

It has been suggested that biomaterials can be divided into different generations depending on their bioactivity and clinical goals. Thus, the first generation were developed solely to avoid harming tissues, and the materials were biologically inert. The second generation were developed for tissue bonding, and the bioactivity developed via surface erosion. The third generation were designed for tissue regeneration, and the bioactivity provided via material biodegradation [1]. Magnesium alloys fit into the third generation, and there are now multiple clinical reports of their successful use [2,3,4].

**Figure 1:**
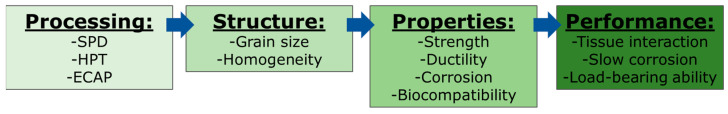
Summary of the relationship between processing, structure, properties and performance of magnesium for biomedical applications.

The performance of a biodegradable implant is linked to its tissue interaction, the type of corrosion and corrosion rate, the load-bearing ability and the reliability of its use. The performance is therefore affected by a combination of multiple properties, including the material strength, ductility, corrosion and biocompatibility. These properties are strongly affected by the material structure which depends on alloy composition and processing operations. It follows that severe plastic deformation (SPD) techniques, such as high-pressure torsion (HPT) and equal-channel angular pressing (ECAP), have attracted significant attention as processing operations, with the ability to improve the properties and performance of magnesium-based implants due to their impact on the grain refinement and homogenization of the structure. These points are illustrated in the processing–structure–properties–performance relationships, as shown in Figure 1.

There has been great interest in evaluating the structure and properties of magnesium alloys processed via SPD. Many advances were obtained in the past twenty years in understanding the mechanism of the grain refinement of magnesium alloys during ECAP, and processing routes were developed enabling the fabrication of magnesium alloys with ultrafine (<1 μm) grain structures. In addition, the HPT technique, which enables the processing of magnesium and its alloys at room temperature and achieves significant grain refinement, gained considerable popularity in recent years. As a consequence, there is now a large number of studies on the use of SPD to process magnesium and its alloys aiming for biomedical applications. These studies reveal trends in the relationship between grain size and mechanical properties, whereas the effect of corrosion is not clear. Various topics, including the processing of Mg, mechanisms of microstructure refinement, mechanical and corrosion properties and the biological response, are critically reviewed in the present paper. 

## 2. Processing by Severe Plastic Deformation

**Figure 2:**
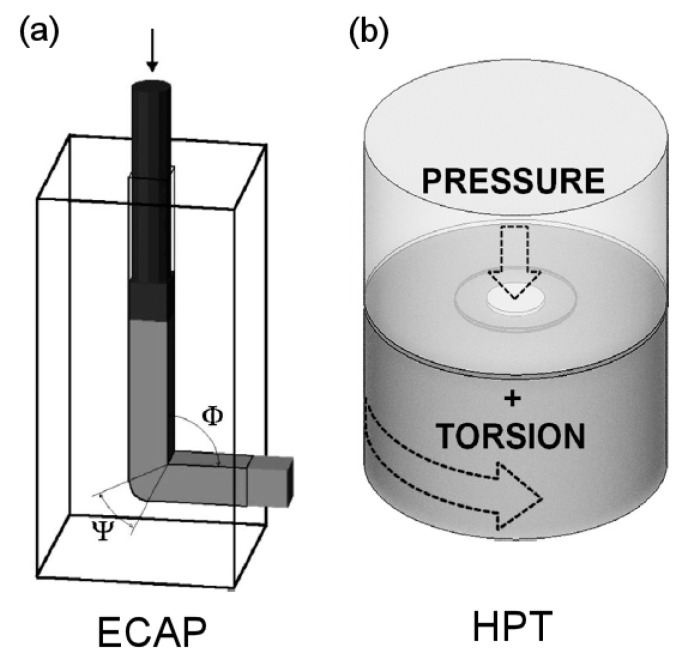
Illustration of the principles of (**a**) ECAP [20] and (**b**) HPT [21].

There are many different SPD techniques, including accumulative roll bonding (ARB), multi-directional forging (MDF), twist-extrusion (TE) and others [5]. Surface treatments such as surface mechanical attrition treatment (SMAT) [6] and ultrasonic shot peening [7] can also induce large amounts of plastic deformation. It is fair to state that ECAP and HPT are the most common techniques used to process magnesium alloys. Therefore, the present paper focuses on these two processing operations, which are illustrated in Figure 2. A billet is pressed through a die containing two intersecting channels of an equal cross-section in ECAP. Shear deformation is imposed in the intersection between the channels, and the amount depends on the geometry of the die. The process can be repeated since the dimensions of the billet remain unchanged after passing through the die. In HPT, a sample in the shape of a disc is compressed between two rigid anvils and subjected to torsion deformation via the rotation of one of the anvils. The amount of deformation introduced during HPT depends on the number of rotations and the dimensions of the sample. The main processing parameters include the temperature, deformation rate, amount of deformation imposed, shear orientation and hydrostatic stress state. The principles of the ECAP and HPT and the influence of processing parameters are summarized in earlier comprehensive reviews [8,9]. 

The initial attempts to process magnesium via ECAP were carried out at very high temperatures, thereby compromising grain refinement due to their low formability at low temperatures. For example, an early report described the processing of pure magnesium and two magnesium alloys via ECAP at temperatures of 473 K and higher, but these high temperatures hindered the grain refinement and, as a consequence, the reported minimum grain sizes were larger than 10 μm [10]. Later studies showed that the tendency for cracking could be reduced by increasing the angle between the channels in the ECAP die [11]. It was also found that a previous thermo-mechanical processing step, such as extrusion, enables ECAP processing at lower temperatures and leads to finer grain sizes [12]. The use of backpressure [13,14,15,16] also enables the processing of magnesium alloys at lower temperatures.

Cracks are avoided during HPT processing due to the large hydrostatic stresses. As a consequence, magnesium and its alloys are readily processed by this technique at room temperature and exhibit significant grain refinement. The grain sizes achieved in magnesium processed via HPT are typically smaller than in their counterparts processed via ECAP. However, it has been shown that structural heterogeneities due to localized deformation might develop in magnesium processed via HPT [17,18]. These heterogeneities may affect the overall properties of the sample. For example, a recent report described localized corrosion in discs of magnesium alloys, which was attributed to the development of localized deformation during HPT processing [19]. 

## 3. Structure Evolution

**Figure 3:**
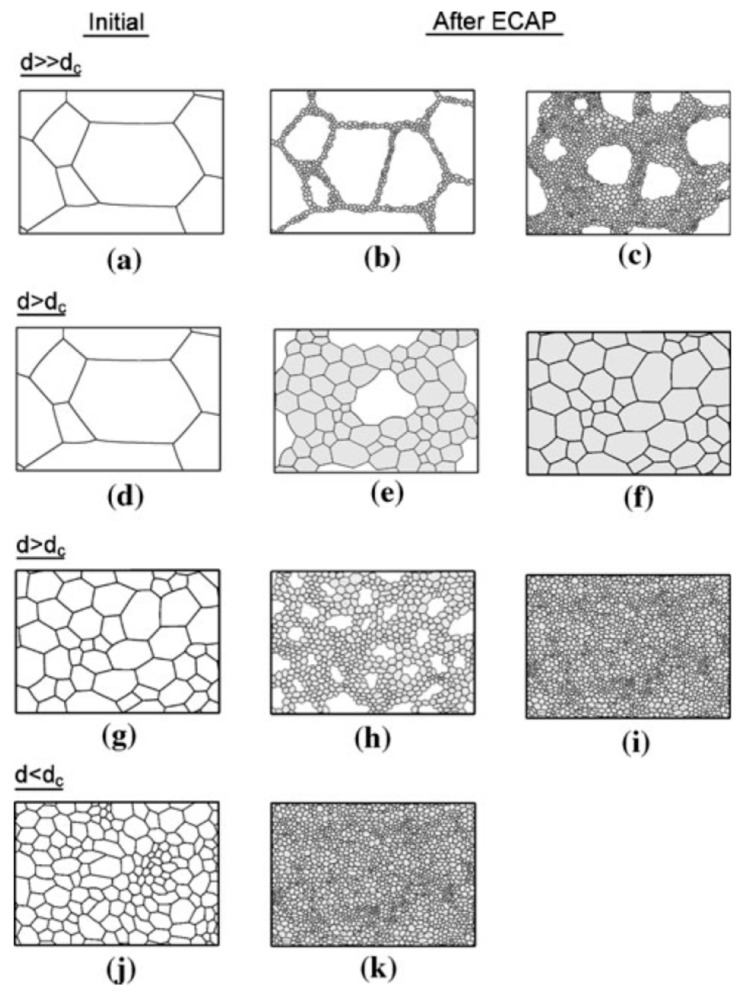
Illustration of the mechanism of grain refinement of magnesium processed via ECAP [23].

The mechanism of grain refinement in magnesium and its alloys during SPD processing differs from the mechanism observed in other metallic materials. The difference is attributed to the hexagonal close-packed (h.c.p.) structure and the occurrence of dynamic recrystallization at high temperatures. A detailed description of the mechanism of recrystallization in magnesium is available elsewhere [22]. Thus, while materials with face-centered cubic (f.c.c.) and body-centered cubic (b.c.c.) structures display homogeneous grain refinement, magnesium displays grain refinement initially concentrated near grain boundaries. A model was developed to illustrate the mechanism of grain refinement in magnesium processed via ECAP [23,24]. Figure 3 shows an illustration of the model. Thus, the grain structure evolution depends on the initial grain size, the ECAP processing conditions (strain rate and temperature) and the amount of strain imposed on the material. The size of the newly formed grains depends strongly on the temperature and strain rate during ECAP. The lower the temperature and the higher the strain rate, the smaller the size of the new grains which are formed along the grain boundaries of the starting material. The difference in size between the initial grains and the new grains and the amount of strain imposed during ECAP will affect the homogeneity in the final structure. Thus, in situations in which the size of the initial grain structure, *d*, is larger than a critical grain size, *d_c_*, the new grains will not occupy the whole volume and a heterogeneous grain size distribution develops.

ECAP processing of coarse-grained magnesium can lead to heterogeneous grain size distribution if the processing is carried out at low temperatures, with this route being illustrated in the top row of Figure 3, or a homogeneous distribution of moderate-sized grains if the processing is carried out at high temperatures, and this is illustrated in the second row. The incorporation of a preliminary thermo-mechanical processing operation can refine the initial structure of the material, and this route, illustrated in the bottom rows, can lead to the development of a homogeneous distribution of fine grains. 

There is experimental evidence in the literature supporting the different grain structure evolutions depicted in Figure 3. It is therefore expected that the grain structures produced via ECAP vary significantly in different reports and that these different structures will display different properties. In fact, there is a great dispersion in the properties of magnesium processed via ECAP in the literature, and this will be examined in the next sections.

**Figure 4:**
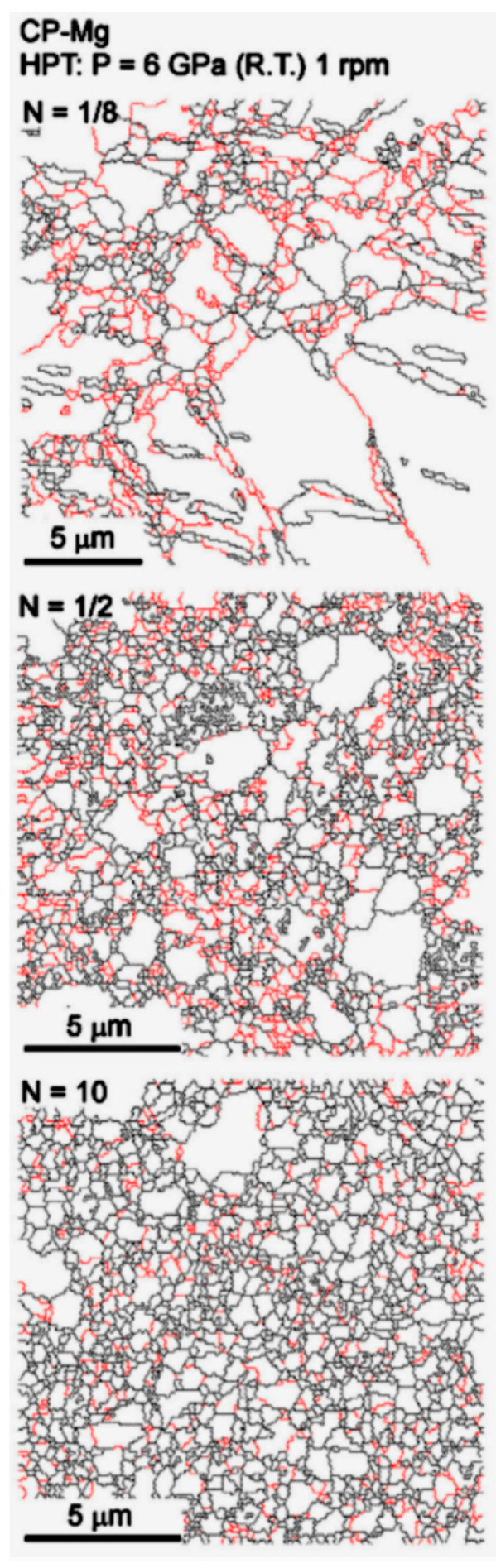
Evolution of the grain structure of pure magnesium during HPT processing [28].

The amount of strain imposed during HPT is typically much larger than in ECAP. This means that, despite some heterogeneity in grain size distribution observed in the early stage of processing, the grain structure usually evolves to a homogeneous distribution of ultrafine grains. Figure 4 shows the evolution of the grain structure, observed using EBSD, of pure magnesium at different stages of HPT processing [25]. High-angle boundaries are depicted in black lines and low-angle boundaries in red. Coarse grains surrounded by fine grains are observed at a low number of turns and a homogeneous distribution of ultrafine grains is observed after multiple turns. As HPT processing is usually carried out at room temperature, grain growth is prevented, and the final grain sizes are smaller than in ECAP. For example, final grain sizes larger than 10 μm were reported in pure magnesium processed via ECAP at high temperatures [10,26], while grain sizes smaller than 1 μm were reported in pure magnesium processed via HPT at room temperature [25,27]. 

**Figure 5:**
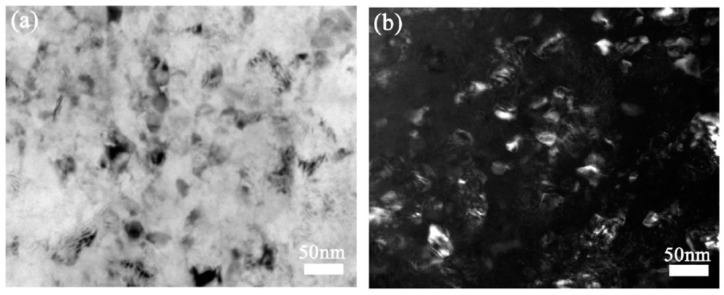
TEM images of the structure of an Mg–8.2Gd–3.8Y–1.0Zn–0.4Zr alloy processed via HPT [30].

The grain sizes achieved during SPD processing can be reduced by the incorporation of alloying elements and second phase particles which reduce grain boundary mobility. Consequently, the average grain sizes reported in magnesium alloys processed via SPD are usually smaller than the grain sizes obtained in pure magnesium. A summary of grain sizes reported in magnesium and its alloys after HPT processing is available elsewhere [29]. Figure 5 shows the structure of an Mg–8.2%Gd–3.2%Y–1.0%Zn–0.4%Zr alloy in which a mean grain size of only 35 nm was reported after processing via HPT [30]. 

## 4. Mechanical Properties

There are multiple reports of higher strength in magnesium and its alloys after ECAP processing and this is attributed to grain refinement. There is also a significant dispersion in the mechanical properties of magnesium processed via ECAP. The dispersion is a consequence of the multiple processing routes which produce different microstructures, as discussed in the previous section. Moreover, the mechanical behavior of magnesium depends strongly on the sample texture and loading direction. The texture developed in magnesium is affected by the number of passes [31,32], the die geometry [33] and the ECAP route [31], which is defined by the sequence of rotations of the billet between successive passes. The alloy composition can also affect the intensity of the texture [32]. 

**Figure 6:**
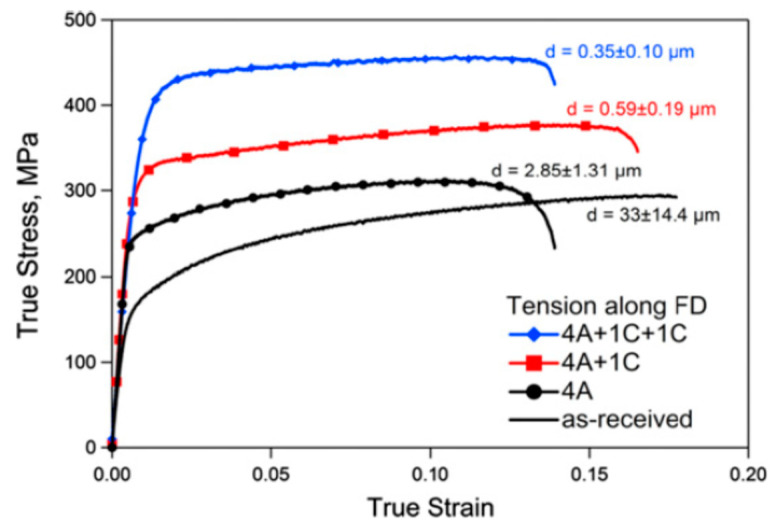
Stress–strain curves of an AZ31 alloy processed via ECAP [37].

The mechanical behavior of magnesium alloys processed via ECAP varies significantly depending on the loading direction [34,35]. Accordingly, ECAP processing might produce a texture meaning basal slip is favored for loading along the billet direction, and this leads to enhanced ductility for tensile tests in this direction [34,36]. Nevertheless, high strength can also be achieved by controlling ECAP processing parameters and texture development. Figure 6 shows tensile stress–strain curves for a magnesium alloy AZ31 processed via ECAP in which the grain size was significantly refined [37]. Increasing strength with decreasing grain size is observed.

**Figure 7:**
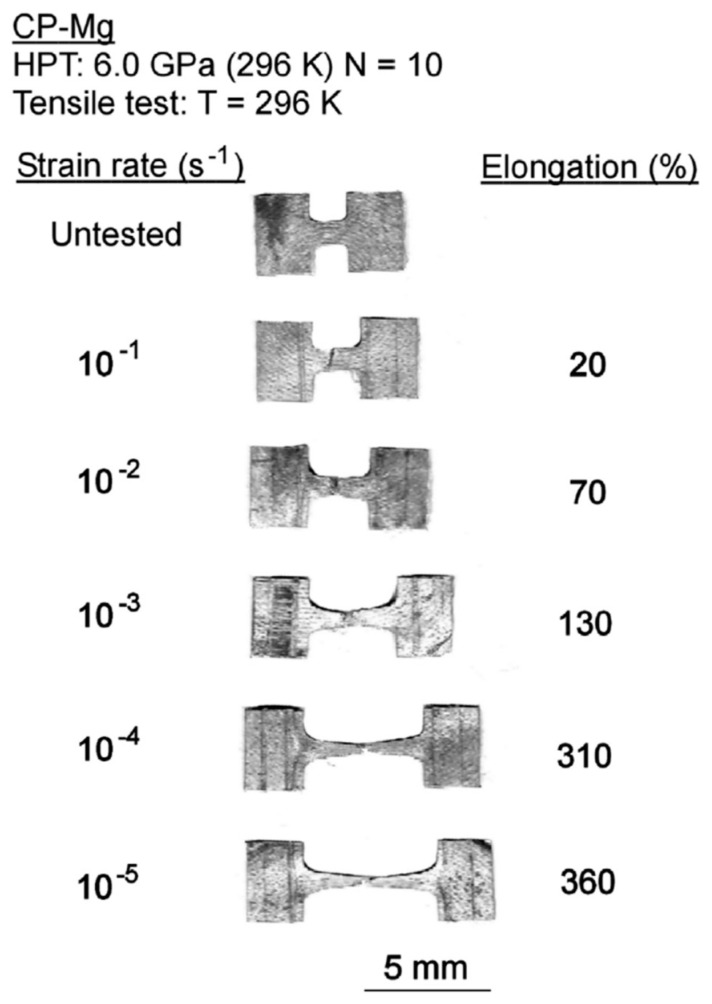
Appearance of specimens of pure magnesium processed via HPT and pulled to failure at room temperature [25].

The grain refinement introduced via SPD processing might also soften magnesium. Pure magnesium displays inverse Hall–Petch behavior at room temperature so that grain refinement below a certain level can decrease its strength [38]. It is interesting to note that the decrease in strength in ultrafine-grained pure magnesium is associated with a remarkable increase in ductility. Figure 7 shows the appearance of tensile samples of pure magnesium processed via HPT and tested at room temperature. The average grain size produced by this SPD technique was only 0.32 μm [25]. Elongations as high as 360% are observed in this material. Such exceptional elongations are attributed to the occurrence of grain boundary sliding in this fine-grained pure magnesium. Recent papers show that exceptional elongations and ductilities may also develop in some fine-grained Mg alloys [39,40,41,42], including superplastic elongations in an Mg–Li alloy processed via HPT [43]. 

The effect of grain size on the mechanical properties of magnesium and its alloys has been recently reviewed, and some trends were revealed [44]. Experimental data on flow stress and elongation from over 300 papers were collected and then plotted as a function of the grain size. The data from samples processed via SPD, including both ECAP and HPT, are incorporated into the analysis. It was reported that the amount of alloying elements affects the strength and ductility of magnesium alloys. Two trends were revealed for different grain size ranges in magnesium alloys. For grain sizes larger than ~4 μm, the deformation is twinning-controlled and the data follow a Hall–Petch relation. However, there is a break in the flow stress relationship (σ, vs. the grain size, *d*) for grain sizes smaller than ~4 μm, and the data follow the relationship predicted by the mechanism of grain boundary sliding [45], which is in good agreement with other metallic materials [46,47,48]. This regime is then characterized as slip-controlled. 

**Figure 8:**
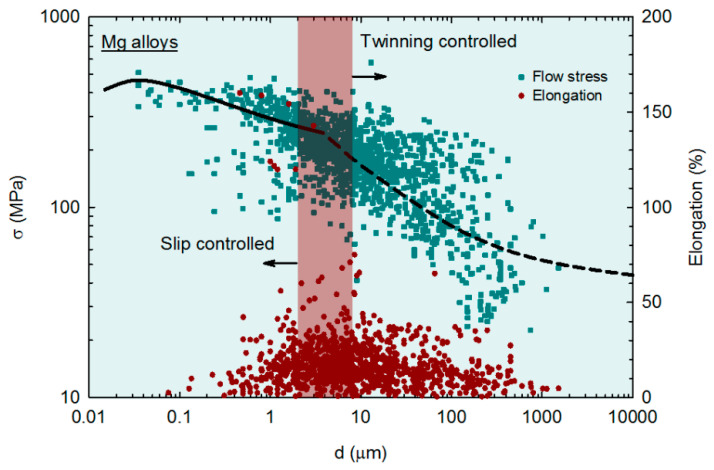
Flow stress and elongation in tension of multiple magnesium alloys plotted as a function of the grain size [44].

The data are shown in Figure 8 and the different deformation regimes are depicted. It follows that the slope between flow stress, σ, and grain size, *d*, is lower in the fine grain regime, which limits the ability of grain refinement strengthening in magnesium alloys. A plateau in strength and inverse Hall–Petch behavior is predicted at very small grain sizes, and this agrees with experimental observations [49,50]. The data for elongation in tension are also plotted in Figure 8 and show different trends. Ductility tends to decrease with increasing grain size in the twinning-controlled regime (coarse grains), and the opposite trend, ductility decreasing with decreasing grain size, is observed in the slip-controlled regime (fine and ultrafine grains). A peak in ductility is observed in the grain size range in which there is a transition between the deformation mechanisms, which is highlighted by a different shade. Pure magnesium and a few alloys are the exceptions to this trend since these materials display increased ductility with decreasing grain size in the slip-controlled regime.

**Figure 9:**
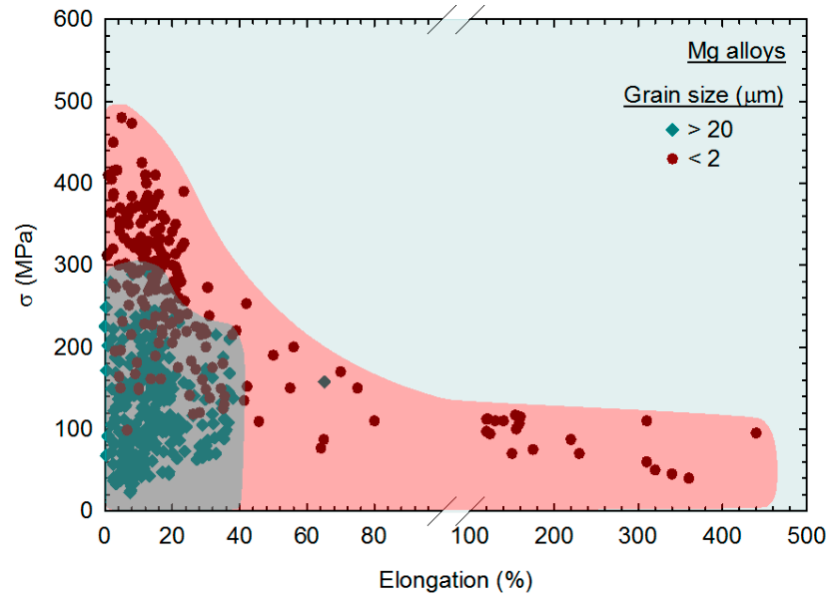
Flow stress (σ) of magnesium alloys plotted as a function of the elongation in tension for samples with different grain size ranges [44].

The experimental data of flow stress of magnesium alloys were also plotted as a function of the elongation in tension to evaluate the strength–ductility relationship [44]. It was reported that grain refinement improves the mechanical properties of magnesium alloys, leading to a significant expansion of range in flow stress, σ, vs elongation plots. This is shown in Figure 9, in which the data from coarse-grained magnesium (*d* > 20 μm) and fine-grained (*d* < 2 μm) are plotted with different symbols. Fine-grained magnesium can display higher strength, a better combination of strength and ductility and exceptional ductility compared to their coarse-grained counterparts [44]. Accordingly, the trends revealed in this analysis show that SPD processing can significantly improve the mechanical properties of magnesium and its alloys due to its ability to promote grain refinement.

## 5. Corrosion Behavior

It has been suggested that grain refinement increases the corrosion resistance of magnesium [51,52,53]. It is expected that severe plastic deformation via ECAP and HPT increases the corrosion resistance, provided a significant structure refinement and homogeneity are introduced. This modification has a favorable effect on corrosion resistance and pitting susceptibility. In a more homogeneous surface, corrosion behavior tends to change from local to uniform and general corrosion. The increasing grain boundary density can lead to an improvement in passive film formation and adhesion. 

A review [54] on the effect of SPD on corrosion behavior concluded that SPD does not compromise corrosion resistance and, in many cases, improves it, despite some contradictory results. There are to date a large number of investigations of the corrosion behavior of magnesium processed via ECAP and HPT, and Table 1 displays the comprehensive summary based on the material, SPD processing method, grain size, mechanical properties, corrosion testing conditions and results. Despite a large number of investigations, it is not easy to establish trends in the corrosion behavior of Mg due to the multiple variables, including alloy composition, type of test and corrosion media, which affect the output. Many papers reported that SPD improves the corrosion resistance of Mg and its alloys, but there are also reports that it does not affect, or it even deteriorates, the corrosion resistance. This divergence is discussed next.

**Figure 10:**
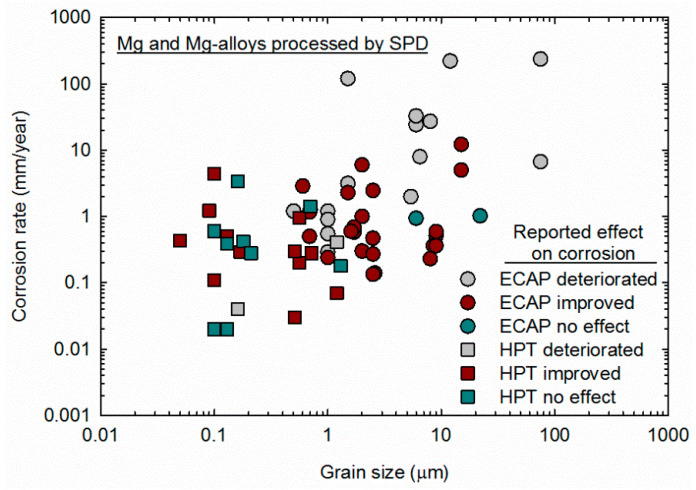
Corrosion rate reported in samples of magnesium processed via ECAP [52,55,56,57,58,59,61,62,63,64,65,66,67,69,71,73,74,75,76,77,78,79,80,82,83,84,85,86,87] and HPT [19,88,90,91,92,93,94,95,97,98,99,100] plotted as a function of the grain size.

It is important to keep in mind that grain size and homogeneity should affect the corrosion behavior. SPD processing via ECAP is usually carried out at high temperatures due to the limited ductility of magnesium and its alloys at lower temperatures. As discussed previously, processing at high temperatures compromises the grain refinement ability, and therefore many papers reported coarse grain structures after ECAP processing. Additionally, the mechanism of grain refinement of magnesium differs from other metallic materials in a way that a heterogeneous grain size distribution might develop after a few passes of ECAP [23,24,101]. Therefore, the results summarized in Table 1 include studies in samples in which SPD processing did not refine the grain structure significantly, and the grain structure is rather heterogeneous. The corrosion rate in samples processed via ECAP and HPT are plotted as a function of the grain size in Figure 10, and different symbols are used to separate the results in which the authors reported that SPD improved corrosion resistance from the results in which SPD did not affect or deteriorated the corrosion resistance. Different symbols are also used to separate results from samples processed via ECAP and samples processed via HPT. 

It is apparent that many studies in which the material was processed via ECAP failed to produce samples with ultrafine (less than 1 μm) grain sizes. Most of the studies reporting that SPD deteriorated corrosion resistance made use of samples in which the grain structure was not ultrafine. Careful observation of the data in Figure 10 shows that decreasing the grain size decreases the corrosion rate, despite the different alloy compositions and testing methods. This observation supports the theory that the grain interior acts as a cathode and the grain boundary as an anode during corrosion and that a decrease in the ratio between the cathode and anode area fraction can decrease the corrosion rate [54,102]. It is also apparent that samples produced via HPT display smaller grain sizes and lower corrosion rates. This is attributed to the lower temperature in which HPT is usually carried out and the larger amount of strain imposed. The former reduces the grain size, and the latter increases the homogeneity of the structure.

It is worth noting that the homogeneity of the structure can significantly affect the corrosion behavior of magnesium processed via SPD. An early paper reported the processing of pure magnesium via ECAP at different temperatures, a process which led to different average grain sizes and grain size distributions. A higher corrosion rate was reported in a sample in which the grain size distribution was less homogeneous [55]. A deteriorated corrosion resistance was reported in a ZK60 magnesium alloy processed by a few turns of HPT and was also attributed to heterogeneous grain size distribution [93]. However, further processing of this alloy to a larger number of turns caused homogenization of the grain structure and improved the corrosion resistance [93]. A recent paper showed that localized corrosion can develop in areas in which deformation heterogeneity takes place during HPT processing. This might include the center and the edge of the discs [19]. Therefore, it is of great importance that SPD processing routes are developed in order to produce homogeneous structures. Moreover, recent papers have shown that residual stresses imposed with surface treatments can affect the corrosion behavior of magnesium alloys [103,104]. Improvement in corrosion resistance has also been reported in magnesium alloys processed via friction stir processing [105] and multi-axial isothermal forging [106].

## 6. Biological Response

It is now well known that magnesium displays a good biological response and there are prospects of increasing its use as an implant material [2,4,107,108]. The previous sections showed that severe plastic deformation can improve both the mechanical properties and the corrosion behavior of this material. However, it is important to review studies on whether such processing could compromise their biological response. Table 2 summarizes the output of cytotoxicity tests carried out in magnesium processed via ECAP or HPT. Different alloys and different cell types were considered in these investigations, and in practice, there is no report of any significant toxicity. In fact, an increase in cell viability and cell proliferation was reported in a WE43 magnesium alloy processed via ECAP compared to the homogenized state [73].

**Figure 11:**
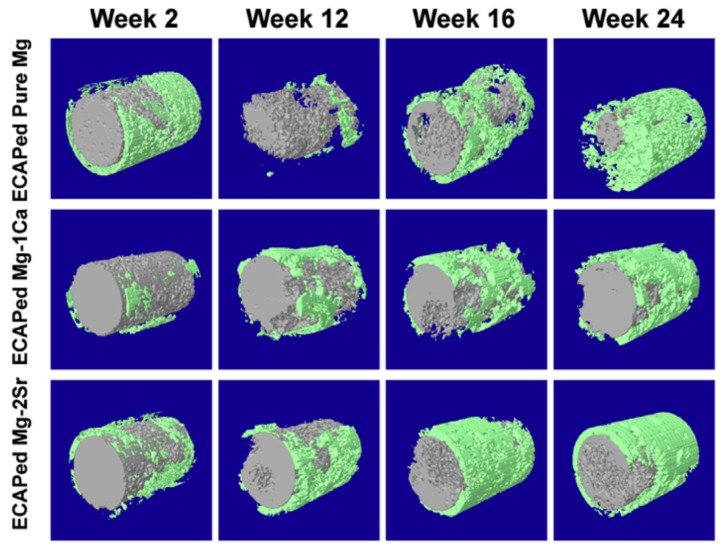
Micro-CT 3D reconstruction of implants (gray in color) of Mg, Mg–1%Ca and Mg–2%Sr processed via ECAP and bone around the implant (green in color) after 2, 4, 16 and 24 weeks of in vivo degradation [58].

In addition to cytotoxicity tests, many studies evaluated the in vivo response of implants produced from magnesium processed via ECAP and HPT. These studies were carried out using rats, mice, rabbits and dogs as animal models, and most of them focused on bone implants. Table 3 summarizes these tests, and it is shown that, generally, the implants from magnesium processed via SPD display good in vivo responses. One of the major concerns regarding the degradation of magnesium in biological applications is the accumulation of hydrogen gas, which is a by-product of corrosion. However, only one of the studies, in which the implants from an Mg–Zn–Ca alloy were inserted subcutaneously, reported the formation of gas under the skin [82]. A good biological response including bone formation around the implant was observed in pure magnesium and Mg–Ca and Mg–Sr alloys processed via ECAP [58] and via HPT [90]. Figure 11 shows the three-dimensional reconstruction of the implants of material processed via ECAP (gray) and the surrounding bone (green). All the materials degrade gradually, maintained the rod shape of the implant during the whole implantation period and no severe local corrosion was observed. After 12 weeks post-operation, the volume of implants remained around 75% of their original volumes, and at 24 weeks, for the ECAP-processed pure Mg group, around ~25% of implant volume remained, while over 50% of implant volume was left for the ECAP-processed Mg–1Ca alloy and Mg–2Sr alloy groups.

## 7. Overall Performance

**Figure 12:**
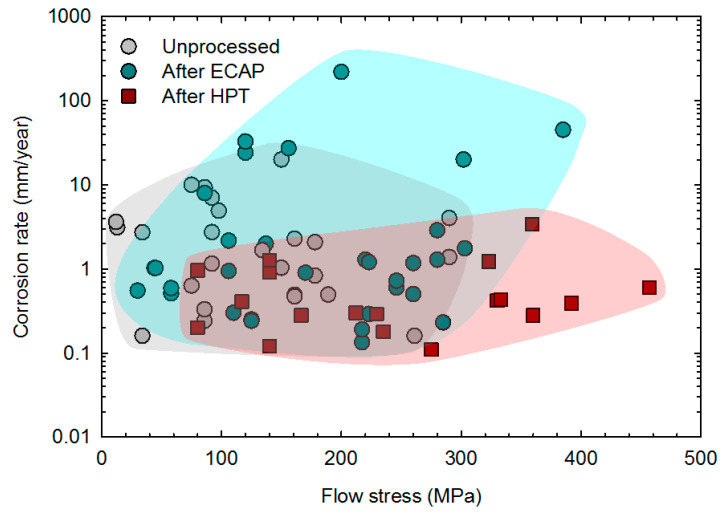
Corrosion rate vs. flow stress of magnesium and its alloys before and after SPD processing. Data from the literature [19,57,58,59,64,66,70,72,76,77,78,79,80,82,83,84,85,86,87,88,90,91,92,94,100].

The previous sections showed that severe plastic deformation can effectively refine the grain structure of magnesium and its alloys, improve the mechanical properties and increase the corrosion resistance without compromising biocompatibility. There are contradictory reports which are mostly related to samples processed via ECAP in which the grain structure was not significantly refined and/or there was heterogeneity in the grain size distribution. The relationship between the corrosion rate and mechanical strength is depicted in Figure 12 for samples of unprocessed material and samples processed via ECAP and HPT. Thus, the data for samples processed via ECAP do not differ notably from the unprocessed material. The range of data suggests a slight increase in strength but a slight increase in corrosion rate as well. There is a notable trend that the data for samples processed via HPT show an improvement in overall performance. The range of data for these samples extends to higher strength with lower corrosion rates. This improved performance in magnesium and its alloys processed via HPT is then attributed to the ability of HPT to promote significant grain refinement and structural homogenization.

**Figure 13:**
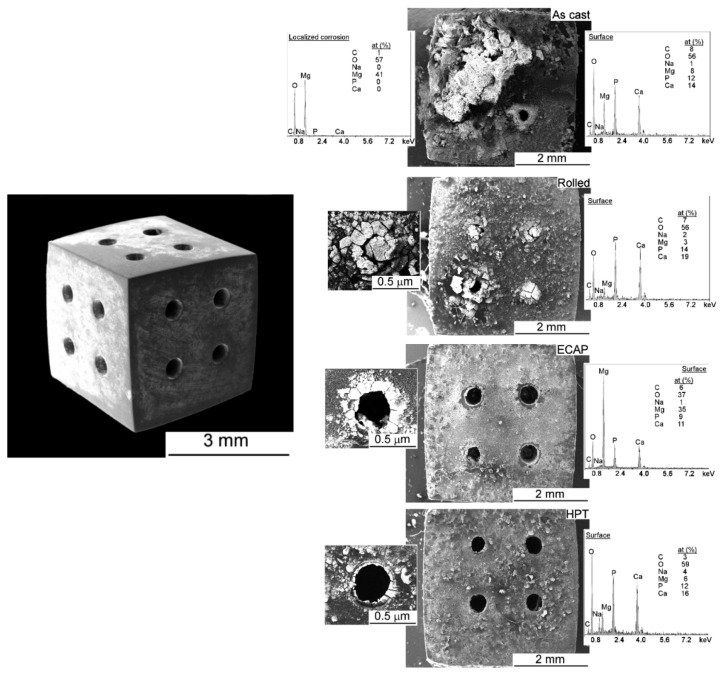
Appearance of scaffolds of pure magnesium with different processing history and immersion in Hank’s solution for 14 days [109].

The positive effect of SPD on corrosion is not limited to a decrease in the average corrosion rate. It is also expected that corrosion becomes uniform [102], and homogeneous corrosion has been reported after HPT processing of an Mg–Zn–Ca alloy [97]. Figure 13 shows the appearance of scaffolds of pure magnesium with different processing histories after immersion in Hank`s solution for 14 days. It was reported that the samples processed via ECAP and HPT exhibited lower corrosion rates and more uniform corrosion, while the as-cast material displayed localized corrosion [109]. 

## 8. Magnesium Composites

The performance of magnesium implants can be improved by the incorporation of other materials into composites [110]. High-pressure torsion provides the opportunity to consolidate different metallic materials, including magnesium, into a bulk sample in which the structure is significantly refined [111,112]. Thus, recent papers have exploited this procedure, and different composites and hybrids have been produced using HPT. For instance, it is now known that the strength of magnesium can be significantly increased by mixing it with other metallic materials such as Al [113,114,115] or Zn [116,117,118,119]. The Mg–Zn system is especially interesting since both materials are biodegradable, and its corrosion behavior in SBF has been investigated [119]. 

**Figure 14:**
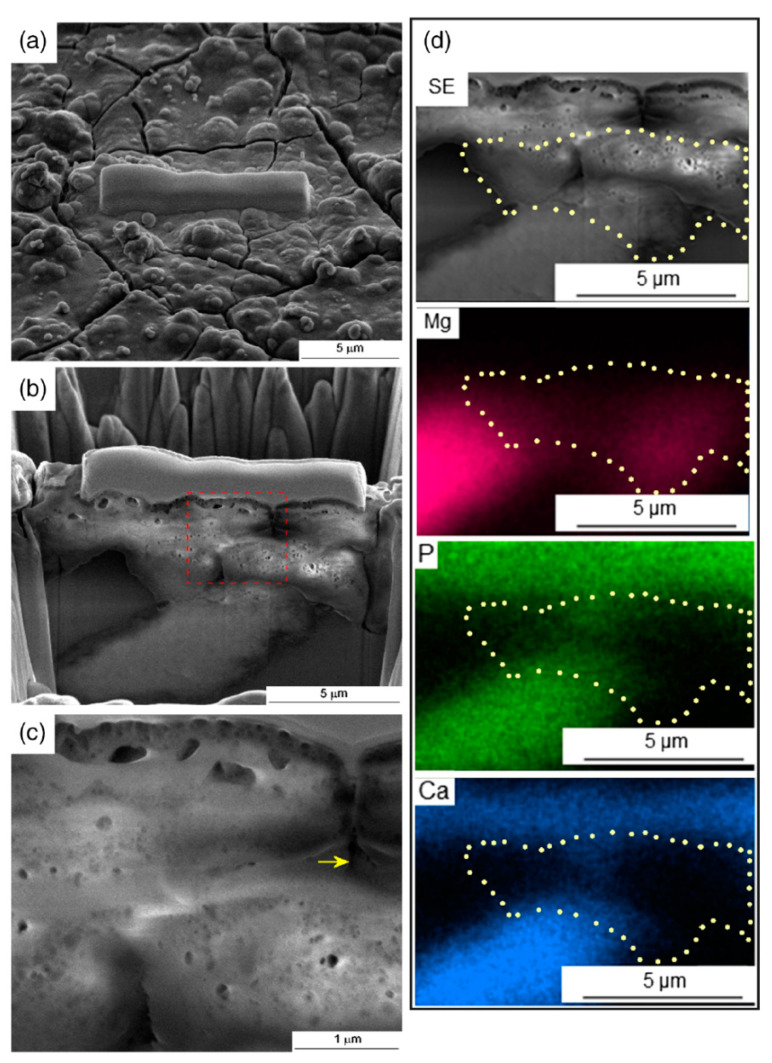
Elemental composition distribution along the depth of an Mg–HA composite produced via HPT and subjected via immersion in Hank’s solution [121].

It is also possible to incorporate hard ceramic particles into a magnesium matrix through HPT, and this provides the opportunity to produce magnesium composites with bioactive particles. Recent papers reported the incorporation of bioactive glass [120] and hydroxyapatite [120,121] into the magnesium matrix. Cytotoxicity tests showed the Mg–HA composite is biocompatible [120]. Figure 14 shows an elemental composition map across a cut on the surface of the composite after immersion in Hank’s solution. A hydroxyapatite particle and the corrosion product layer are rich in Ca and P, and it was reported that the corrosion product layer is uniformly distributed on the surface of the sample [121]. It is expected that such a surface layer improves the interaction between a magnesium implant and the surrounding tissue. 

## 9. Summary and Conclusions

The structural features, mechanical properties, corrosion behavior and biological response of magnesium and its alloys subjected to severe plastic deformation are critically reviewed. The following trends were highlighted.

1. The unique mechanism of grain refinement of magnesium subjected to ECAP processing is associated with a dispersion in grain structure which includes a broad range of average grain sizes and grain size distributions. Processing via HPT is more effective than other processing techniques for grain refinement and structure homogenization, although there are some reports of localized deformation.
2. The mechanical properties of magnesium and its alloys are significantly improved via grain refinement. High strength and exceptional ductility are observed in fine and ultrafine-grained magnesium processed via SPD.
3. There seems to be a trend of increased corrosion resistance with decreasing grain size in magnesium and its alloys. Most of the studies report improved corrosion resistance after SPD processing. The reports of decreased corrosion resistance after SPD are mostly related to samples processed via ECAP in which the grain structure was not significantly refined and/or the grain structure was heterogeneous.
4. Biocompatibility tests and in vivo investigations reveal no detrimental effect of SPD processing on the biological response of magnesium.
5. The best combinations of improved strength and corrosion resistance are observed in magnesium and alloys processed via HPT. There are also reports of a reduced tendency for localized corrosion in magnesium processed via HPT.
6. High-pressure torsion can also be used to produce magnesium-based composites with improved strength and with the incorporation of bioactive particles.
7. Overall, the present review shows that care must be taken during SPD processing in order to attain a homogeneous structure with ultrafine grains in magnesium. Future research in this field should evaluate the degree of homogeneity of the structure at different locations of the processed material and relate this information to mechanical properties and corrosion behavior.

## Figures and Tables

**Table 1:** Summary of magnesium alloys, SPD processing method, grain size (d), flow stress (σ), elongation to failure (El.), corrosion rate (C.R.), corrosion media, corrosion tests and reported SPD effect on corrosion resistance.

Material	Method	D (µm)	σ (MPa)	El. (%)	C.R. (mm/yr)	Media *	Corrosion Test **	SPD Effect on Corrosion Resistance	Reference
Pure Mg	As cast	1500	12	5.9	3.62	SBF	E.P.	Improved	[55]
	ECAP (4 passes)	365	37	8.1	0.13		W.L.		
					0.09		E.P.		
		65	48	10.3	0.38		W.L.		
					0.32		E.P.		
		30	51	16.5	0.73		W.L.		
					0.67		E.P.		
		9	58	15.9	0.59		W.L.		
					0.36		E.P.		
Pure Mg	As cast	125.00			0.24	0.1 M NaCl	E.P.	Improved	[52]
	ECAP (1 pass)	25.00			0.19				
	ECAP (8 passes)	2.60			0.14				
Pure Mg	As cast	1150.00			12.60	3.5 wt%. NaCl	W.L.	Deteriorated	[56]
					1.14		E.P.		
	ECAP (1 pass)	150.00			121.00		W.L.		
					2.74		E.P.		
	ECAP (6 passes)	75.00			236.00		W.L.		
					6.17		E.P.		
Pure Mg	As cast	1500.00	13	5.9	3.12	HS	E.P. / H.E.	Improved	[57]
	ECAP (4 passes)	9.00	58	15	0.51				
Pure Mg	ECAP (6 passes)	1.00	30	12	0.55	In Vivo	μ-T.	Deteriorated	[58]
Mg-1%Ca	ECAP (6 passes)	1.00	125	7	0.24			Improved	
Mg-2%Sr	ECAP (6 passes)	2.00	110	8	0.30			Improved	
Pure Mg	As cast	250.00	44	4.4	1.02	PBS	E.P.	No significant effect	[59]
	ECAP (6 passes)	22.00	45	10.3	1.02	PBS	E.P.		
ZK60	Extruded	2~15	290	15	1.38	PBS	E.P.	Improved	
	ECAP (4 passes)	0.70	220	33	1.28	PBS	E.P.		
AE21	ECAP (8 passes)	2.50				0.1 M NaCl	E.I.	Deteriorated	[60]
AE42	ECAP (8 passes)	2.50						Improved	
AE42	Extruded	4.50			2.54	KSBF	Atomic Absorption Spectrometry	Improved	[61]
AE42	ECAP (8 passes)	1.50			2.29				
AZ31	Squeeze cast	450.00			0.32	HS	W.L.	Improved	[62]
	ECAP (4 passes)	2.5			0.27				
AZ31	Extruded	28.00			2.09	HS	E.P.	Improved	[63]
	ECAP (4 passes)	8.5			0.38				
	ECAP-BP (4 passes)	1.70			0.58				
AZ31	As cast	30.00			125.00	3.5 wt%. NaCl	E.P.	Deteriorated	[64]
	ECAP (4 passes)	12.00	200		220.00				
AZ31	Annealed	47.00			18.00	SBF	W.L.	Improved	[65]
					1.6	In Vivo			
	ECAP (4 passes)	1~5			6.00	SBF			
					1.1	In Vivo			
AZ31	As received	27.5	97.7	14.2	4.92	KSBF	E.P./H.E.	Deteriorated	[66]
	ECAP (1 pass)	8.3	122.7	22.8	1.91				
	ECAP (2 passes)	6.8	109.5	36.0	6.54				
	ECAP (4 passes)	6.5	86	46.8	7.97				
AZ91	ECAP (12 passes)	1.50			120	3.5 wt%. NaCl	W.L.	Deteriorated	[67]
					3.15		E.P.		
LAE442	ECAP (12 passes)	1.5				0.1 M NaCl	E.P.	Improved	[68]
LAE 442	Extruded	21.00			0.80	KBM	W.L.	Improved	[69]
					0.92	MEM			
	ECAP (12 passes)	1.7			0.59	KBM			
					0.69	MEM			
WE43	Extruded	9.1 [70]	189	20.9	0.494	Hank’s solution	E.P.	Improved	[71]
	ECAP (1 pass)	6 [70]	245	15	0.15				
	ECAP (2 passes)	8 [70]	285	14	0.23				
WE43	As cast	135.00	170	9.70		In Vivo		Improved	[72]
	ECAP (4 passes + extrusion)	50.00	225	12.2					
WE43	Homogenized	65	161	9	0.49	0.9% NaCl	E.P.	Improved	[73]
					2.29		W.L.		
	ECAP (12 passes)	0.69	260	13.2	0.50		E.P.		
					1.17		W.L.		
ZE41A	ECAP (60 passes)	2.5			2.46	DMEM solution	H.E.	Improved	[74]
					0.47		E.P.		
ZE41	As cast	48.00			24.70	1 M NaCl	E.P.	Improved	[75]
					3.00	0.1 M NaCl			
	ECAP (6 passes)	15.00			12.20	1 M NaCl			
					5.00	0.1 M NaCl			
ZFW MP	Extruded	5.00	261	8.4	0.16	HS	E.P.	Deteriorated	[76]
	ECAP (1 pass)		269	2.3	1.13				
	ECAP (3 passes)	0.50	291	11.4	1.2				
	ECAP (4 passes)		303	5.8	1.76				
ZK60	Extruded	1~20	290	18	4.03	PBS	W.L.	Improved	[77]
					1.38		E.P		
	ECAP (4 passes)	0.6	280	30	2.88		W.L.		
					1.28		E.P		
ZM21	As rolled	45.00	150	20	1.03	HS	E.P.	Deteriorated	[78]
	ECAP (1 pass)	18.4	136	21	3.34				
	ECAP (2 passes)	10.9	154	22	1.08				
	ECAP (3 passes)	5.0	128	23	1.28				
	ECAP (4 passes)	5.4	137	27	1.99				
Mg-2.9Gd-1.5Nd-0.3Zn-0.3Zr	AS cast	40.00	85.8	11.8	0.24	SBF	W.L.	Improved	[79]
					0.33		E.P.		
	ECAP (4 passes)	2.50	217.3	18.5	0.13		W.L.		
					0.19		E.P.		
Mg-4.7% Gd-1.42% Nd-0.59% Zn-0.37% Zr	Homogenized	80.00	125	22.7	0.25	HS	H.E.	Deteriorated but changed pitting corrosion to uniform corrosion	[80]
					0.09		E.P.		
	ECAP (4 passes)	1.50	215	30.1	0.80		H.E.		
					0.19		E.P.		
	ECAP (8 passes)	1.00 ***	223	36.2	1.20		H.E.		
					0.29		E.P.		
Mg-1.0%Zn-0.3%Ca	As received (Homog. + Extr.)	106.00	92	13		0.9 wt%. NaCl	W.L.	No significant effect	[81]
	ECAP (4 passes)	6.00	106	24					
Mg-1.0%Zn-0.3%Ca	As received (Homog. + Extr.)	106.00	92	13	1.15	FBS	W.L.	No significant effect	[82]
					2.74	0.9 wt%. NaCl	E.P.		
	ECAP (4 passes)	6.00	106	24	0.94	FBS	W.L.		
					2.17	0.9 wt%. NaCl	E.P.		
Mg-2%Zn-0.5%Mn-1%Ca-1.35%Ce	As cast	60.00	75	4.7	0.63	HS	E.P.	Deteriorated	[83]
	ECAP (12 passes)	1 ***	170	12.5	0.90				
Mg-4%Zn-1%Mn	Homogenized	260.00	92	5.8	7.03	HS	E.P.	Deteriorated	[84]
	ECAP (1 passes)	64.00	117	7.5	14.38				
	ECAP (2 passes)	40.00	124	16.5	17.79				
	ECAP (3 passes)	12.00	174	18	20.38				
	ECAP (4 passes)	8.00	156	21	27.19				
Mg-4%Zn-1%Si	Homogenized	210.00	86	7	9.08	SBF	E.P.	Deteriorated	[85]
					9.34		H.E.		
	ECAP (1 pass)	44.00	105	8.2	9.84		E.P.		
					10.90		H.E.		
	ECAP (2 passes)	20.00	109	8.7	14.02		E.P.		
					14.55		H.E.		
	ECAP (3 passes)	12.00	126	9.4	23.75		E.P.		
					29.81		H.E.		
	ECAP (4 passes)	6.00	120	12	24.14		E.P.		
					32.70		H.E.		
Mg-4.71%Zn-0.6%Ca	As cast	54.5	178	6.2	0.83	HS	W.L.	Improved	[86]
					2.08		E.P.		
	ECAP (4 passes)	1.6	246	11.3	0.60		W.L.		
					0.72		E.P.		
Mg-6%Zn	Homogenized		75	26	10.00	0.9 wt%. NaCl	H.E.	Deteriorated	[87]
	ECAP (4 passes)		302	0.7	20.00				
Mg-12%Zn	Homogenized	150.00	150	19	20.00				
	ECAP (4 passes)		385	0.6	45.00				
Pure Mg	As cast	480.00	34	5	0.16	3.5 wt%. NaCl	E.P.	Improved	[88]
					2.72		H.E.		
	ECAP (4 passes)	3.20	140	8	0.91		E.P.		
					1.26		H.E.		
	HPT (N = 10)	0.56	80	130	0.20		E.P.		
					0.96		H.E.		
Pure Mg	As cast		18	46	-	3.5 wt%. NaCl	E.I.	No significant effect	[89]
	HPT (N = 5)	2.00	142	38	-				
Pure Mg	HPT (N = 5)	1.20	116.9	29.2	0.41	In vivo	μ-T.	Deteriorated	[90]
Mg-1%Ca	HPT (N = 5)	0.17	229.4	1.6	0.29			Improved	
Mg-2%Sr	HPT (N = 5)	0.72	166.4	2.6	0.28			Improved	
Pure Mg	As cast	1000.00			0.03	HS	E.P.	Improved	[91]
	HPT (N = 10)	0.51			0.03				
AZ31	Extruded	16.00			0.02			No significant effect	
	HPT (N = 10)	0.13			0.02				
AZ91	Solution-Treated	110.00			0.01			No significant effect	
	HPT (N = 10)	0.10			0.02				
ZK60	Extruded	2.9			0.22			Deteriorated	
	HPT (N = 5)	0.16			0.04				
Pure Mg	HPT (N = 5)		140		0.12	HS	μ-T.	No effect	[19]
Mg-1% Zn		1.30	235		0.18				
Mg-1% Zn-0.5% Ca			223						
Mg-4% Li-1% Y		0.18	330		0.42				
Mg-8% Li-1% Y		0.21	360		0.28				
WE43			275		0.11				
Pure Mg	As cast	1000.00			0.4	3.5 wt%. NaCl	E.P.	Improved	[92]
	HPT (N = 10)	0.51	212		0.30				
AZ31	Extruded	16.00			0.37			No significant effect	
	HPT (N = 10)	0.13	392		0.39				
AZ91	Solution-Treated	110.00			0.8			No significant effect	
	HPT (N = 10)	0.10	457		0.60				
ZK60	Extruded	2.9			2.5			No significant effect	
	HPT (N = 5)	0.16	359		3.40				
ZK60	Extruded	-			1.32	0.1 M NaCl	H.E.	Improved (after 20 turns)	[93]
	HPT (N = 5)	0.7			1.41				
WE43	Homogenized	65.00	161	9.0	0.47	0.9 wt%. NaCl	E.P.	Improved	[94]
	HPT (200 °C, N = 10)	0.05 ***	333	1	0.43				
	HPT (200 °C, N = 10) + T.T. (200 °C/2 h)	0.07	383	1	1.16			Deteriorated	
Mg-1%Ca	As cast	42.00			1.18	Ringer’s solution	E.P.	Improved	[95]
	HPT (N = 10)	0.10			0.11				
	HPT (N = 10) + T.T. (250 °C/6h)	1.1			0.07			Improved	
Mg-0.45%Zn–0.45%Ca	HPT (N = 10)	1.70				PBS	E.I.	Deteriorated	[96]
Mg-2%Zn-0.24%Ca	As cast	97.00			12.11	KSBF	E.P.	Improved	[97]
	HPT (N = 5)	1.2			0.07				
Mg-2%Zn-0.24%Ca	As cast	11.00			3.92	SBF	E.P.	Improved	[98]
	HPT (N = 5)	0.13			0.50				
Mg-2%Zn-0.24%Ca	Solution-Treated				5.90	SBF	H.E.	Improved	[99]
	HPT (N = 5)	0.10			4.40				
	HPT (N=5) + T.T (210 °C/30 min)	0.31			1.80				
Mg-1%Zn-0.2%Ca	Homogenized	270.00	134		1.67	Ringer’s	E.P.	Improved	[100]
	HPT (N = 10)	0.090	323		1.22				
	HPT (N = 10) + T.T. 200 °C	0.24	327		1.12				
	HPT (N = 10) + T.T. 250 °C	0.55	212		1.04				
	HPT (N = 10) + T.T. 300 °C	4.00	196		1.01				

* Corrosion media: Phosphate Buffer Solution (PBS), Simulated Body Fluid (SBF), Kokubo’s Simulated Body Fluid (KSBF), Hank’s solution (HS). ** corrosion test type: electro-chemical polarization (EP), electro-chemical impedance (EI), weight loss (WL), hydrogen evolution (HE), micro-tomography (μ-T). *** estimated grain size.

**Table 2:** Summary of in vitro tests of cytotoxicity carried out in Mg and Mg alloys processed via SPD.

Material	SPD Process	d (μm)	Test	Cell Type	Time (Days)	Result	Ref.
Pure Mg	ECAP	3.2	Cytotoxicity Cell viability	Human osteosarcoma cell line (SAOS-2)	1	No significant alterations in their mitochondrial metabolic activity.	[88]
			Live/Dead			The cells exposed preserved a vital status.	
Pure Mg	ECAP	0.5~1.5	Cytotoxicity Cell viability	Pre-osteoblasts MC3T3-E1 and human mesenchymal stem cells (hMSC)	5	Cell viability was near or exceed 80%.	[58]
Mg-1%Ca		1				Cell viability was near or exceed 80%.	
Mg-2%Sr		2				Cell viability was near or exceed 80%	
AZ31	ECAP	1.7	Cytotoxicity Cell viability	MG63 cells	3	Cell viability over 70% in the sample with 4 passes of ECAP. Slightly lower viability was observed in the sample with only 3 passes.	[63]
AZ31	ECAP	1~5	Cytotoxicity Cell viability	Rat skeletal muscle (L6) cells	3	Cell viability was near or exceed 80%	[65]
LAE 442	ECAP	1.7	Cytotoxicity Cell viability	L929 cells (murinefibro-blasts)	4	The cell viability was over 70%	[69]
WE43	ECAP	0.69	Cytotoxicity Cell viability	Mouse white blood cells	1	Improved cell viability compared to the initial state.	[73]
			Hemolysis	Mouse red blood cells	1	No significant effect compared to the initial state.	
			Cell proliferation	Mouse Multipotent mesenchymal stromal cells (MMSCs)	7	Improved cell proliferation compared to the initial state.	
ZM21	ECAP	5.4	Cytotoxicity Cell viability	Human osteoblast-like cells (MG63)	3	The cell viability was over 99%.	[78]
			Live/Dead			Large number of living cells were found.	
Mg-1% Zn-0.3% Ca	ECAP	4~8	Cytotoxicity Cell viability	Mouse mononuclear leucocytes (ML)	1	No statistically proven hemolysis and cytotoxic effects.	[82]
			Cell adhesion	Mouse Multipotent mesenchymal stromal cells (MMSCs)	7	Exceeded 100% of adhesion.	
			Cell proliferation		7	Decrease in cell proliferation compared to control.	
			Osteogenic differentiation		21	Osteoinductive activity increased 14% compared to control.	
Pure Mg	HPT	0.56	Cytotoxicity Cell viability	Human osteosarcoma cell line (SAOS-2)	1	No significant alterations in their mitochondrial metabolic activity.	[88]
			Live/Dead			The cells exposed preserved a vital status.	
Pure Mg	HPT	0.59~1.8	Cytotoxicity Cell viability	MC3T3-E1 cells and humanmesenchymal stem cells (hMSCs)	5	Exceeded 80% of cell viability.	[90]
Mg-1%Ca		0.171				Exceeded 80% of cell viability.	
Mg-2%Sr		0.72				Exceeded 80% of cell viability.	
Pure Mg	HPT	0.51	Cytotoxicity Cell viability	Human osteosarcoma cell line (SAOS-2)	1	The cell metabolic activity was over 80%.	[91]
			Live/Dead			Most of the cells exposed preserved a vital status.	
AZ31		0.13	Cytotoxicity Cell viability			The cell metabolic activity was over 80%.	
			Live/Dead			Most of the cells exposed preserved a vital status.	
AZ91		0.10	Cytotoxicity Cell viability			The cell metabolic activity was over 80%.	
			Live/Dead			Most of the cells exposed preserved a vital status.	
ZK60		0.16	Cytotoxicity Cell viability			The cell metabolic activity was over 80%.	
			Live/Dead			Most of the cells exposed preserved a vital status.	

**Table 3:** Summary of in vivo tests carried out in Mg and Mg alloys processed via SPD.

Material (Process)	Animal Model	Number of Days Implanted	Implant Design	In Vivo Corrosion	Findings	Ref.
Pure Mg (HPT)	Rats Femur lateral epicondyle	24 weeks	Cylindrical rods	0.41 mm/year	New bone formed around the surface of implant. Good biocompatibility.	[90]
Mg–1%Ca (HPT)	Rats Femur lateral epicondyle	24 weeks	Cylindrical rods	0.29 mm/year	New bone formed around the surface of implant. Good biocompatibility.	
Mg–2%Sr (HPT)	Rats Femur lateral epicondyle	24 weeks	Cylindrical rods	0.28 mm/year	New bone formed around the surface of implant. Good biocompatibility.	
Pure Mg (ECAP)	Rats Femur lateral epicondyle	24 weeks	Cylindrical rods	0.55 mm/year	At 24 weeks, around ~25% implant volume remained. Good osseointegration.	[58]
Mg–1%Ca (ECAP)	Rats Femur lateral epicondyle	24 weeks	Cylindrical rods	0.24 mm/year	At 24 weeks, around 50% implant volume remained. Good osseointegration.	
Mg–2%Sr (ECAP)	Rats Femur lateral epicondyle	24 weeks	Cylindrical rods	0.30 mm/year	At 24 weeks, around 50% implant volume remained. Good osseointegration.	
AZ31 (ECAP)	Rabbits Femoral bone	60 days	Thin plates	1.1 mm/year	No indication of the hydrogen accumulation and new bone formed. The presence of mild inflammatory response indicates that the material used is biocompatible.	[65]
Mg–Zr–Y–Nd–La (ECAP + Extrusion)	Dog Femur bone	12 weeks	Screws	ECAP reduced the corrosion rate.	A mild inflammatory response in comparison with the unprocessed sample. No sign of hydrogen accumulation and no harmful health effects on the animal bod. New bone formed.	[72]
Mg–1%Zn-0.3%Ca (ECAP)	Mice subcutaneously	4 weeks	Thin plates	20% mass loss in 2 weeks.	Rapid biodegradation of the samples. Significant volume of gas released under the skin near the implant. Penetration of adjacent tissues by crystals of biodegradation products.	[82]

## Data Availability

No new data were created or analyzed in this study. Data sharing is not applicable to this article.
